# Outcome and treatments of dogs with aortic thrombosis: 100 cases (1997‐2014)

**DOI:** 10.1111/jvim.15874

**Published:** 2020-08-15

**Authors:** Mackenzie Ruehl, Alex M. Lynch, Therese E. O'Toole, Bari Morris, John Rush, C. Guillermo Couto, Samantha Hmelo, Stacey Sonnenshein, Amy Butler, Julien Guillaumin

**Affiliations:** ^1^ MedVet Hilliard Hilliard Ohio USA; ^2^ Department of Clinical Sciences, College of Veterinary Medicine North Carolina State University Raleigh North Carolina USA; ^3^ Department of Clinical Sciences Cummings School of Veterinary Medicine at Tufts University North Grafton Massachusetts USA; ^4^ VCA Shoreline Veterinary Referral and Emergency Center Shelton Connecticut USA; ^5^ Couto Veterinary Consultants Hilliard Ohio USA; ^6^ Urban Animal Hospital Seatle Washington USA; ^7^ OakVet Animal Specialty Hospital Oakland California USA; ^8^ Critical Consults, LLC Pittsburgh Pennsylvania USA; ^9^ Department of Clinical Sciences, College of Veterinary Medicine and Biomedical Sciences Colorado State University Fort Collins Colorado USA

**Keywords:** canine, protein‐losing nephropathy, saddle thrombus, thromboembolism, thrombus

## Abstract

**Background:**

Aortic thrombosis (ATh) is an uncommon condition in dogs, with limited understanding of risks factors, outcomes, and treatments.

**Objectives/Hypothesis:**

To describe potential risk factors, outcome, and treatments in dogs with ATh.

**Animals:**

Client‐owned dogs with a diagnosis of ATh based on ultrasonographic or gross necropsy examination.

**Method:**

Multicentric retrospective study from 2 academic institutions.

**Results:**

One hundred dogs were identified. Anti‐thrombin diagnosis, 35/100 dogs were nonambulatory. The dogs were classified as acute (n = 27), chronic (n = 72), or unknown (n = 1). Fifty‐four dogs had at least one comorbidity thought to predispose to ATh, and 23 others had multiple comorbidities. The remaining 23 dogs with no obvious comorbidities were classified as cryptogenic. Concurrent illnesses potentially related to the development of ATh included protein‐losing nephropathy (PLN) (n = 32), neoplasia (n = 22), exogenous corticosteroid administration (n = 16), endocrine disease (n = 13), and infection (n = 9). Dogs with PLN had lower antithrombin activity than those without PLN (64% and 82%, respectively) (*P* = .04). Sixty‐five dogs were hospitalized with 41 subsequently discharged. Sixteen were treated as outpatient and 19 euthanized at admission. In‐hospital treatments varied, but included thrombolytics (n = 12), alone or in combination with thrombectomy (n = 9). Fifty‐seven dogs survived to discharge. Sixteen were alive at 180 days. Using regression analysis, ambulation status at the time of presentation was significantly correlated with survival‐to‐discharge (*P* < .001).

**Conclusions/Clinical Importance:**

Dogs with ATh have a poor prognosis, with nonambulatory dogs at the time of presentation having worse outcome. Although the presence of comorbid conditions associated with hypercoagulability is common, an underlying cause for ATh was not always identified.

**Abbreviations**
ATE: aortic thromboembolism
ATh: aortic thrombosis
DVT: deep venous thrombosis
TPA, tissue plasminogen activator; PLN: protein losing nephropathy

## 1 INTRODUCTION

Aortic thrombosis (ATh) is an uncommon condition in dogs, thought to be associated with a regional prothrombotic environment in the distal aorta.^1^, ^2^, ^3^ This is in contrast to aortic thromboembolism (ATE) in cats, where thrombi formed in the left atria or auricle embolize to the aorta.^4^ Also in contrast with ATE in cats, overwhelmingly because of an underlying cardiomyopathy^4^, ^5^, a variety of diseases have been implicated in ATh in dogs, including protein‐losing diseases, endocrinopathies, and neoplasia.^1^, ^2^, ^3^, ^6^, ^7^, ^8^, ^9^, ^10^, ^11^, ^12^, ^13^, ^14^ In a previous study, 58% of dogs with ATh have no identified underlying disease,^1^ suggesting that ATh with no identifiable cause (or cryptogenic) might be common. Despite an overall guarded prognosis for dogs with ATh, long‐term survival occurs in some dogs, suggesting heterogeneity in the clinical presentation of affected dogs.^2^

Consensus guidelines on the optimal strategy for the prevention and treatment of thrombosis in animals have been established, but never studied prospectively.^15^ In previous studies, treatment strategies have been clinician‐dependent and the variations in cases and treatments make comparison in outcomes difficult. Commonly reported treatment for ATh in dogs typically involves combinations of antiplatelet (eg, clopidogrel, aspirin) alone or in combination with anticoagulant therapies such as heparin, warfarin, direct oral anticoagulants such as rivaroxaban and apixaban^16^, ^17^, ^18^ with thrombolysis by tissue plasminogen activator (TPA) being attempted with varying clinical outcomes.^19^, ^20^, ^21^, ^22^ The efficacy of these therapies in dogs with ATh has been minimally documented.^1^, ^3^

Available literature on ATh in dogs is limited to case reports, descriptive studies on small numbers of dogs, and a few larger studies without information on outcome or treatments.^1^, ^2^, ^3^, ^6^, ^7^, ^8^, ^9^, ^10^, ^11^, ^12^, ^13^, ^14^, ^23^, ^24^, ^25^, ^26^, ^27^, ^28^ The aim of our study was to describe the presenting clinical characteristics of dogs with ATh, specifically underlying causes and descriptions of clinical management.

## 2 MATERIALS AND METHODS

The electronic medical records at Cummings School of Veterinary Medicine at Tufts University (TCSVM) and The Ohio State University College of Veterinary Medicine (OSUCVM) were searched for dogs diagnosed with ATh. Cases were collected between 1997 and 2014 at TCSVM and 2005 to 2011 at OSUCVM. Dogs were included if a diagnosis of a distal ATh was determined by either visualization of a thrombus (ie, a discrete lesion affecting blood flow in the aorta distal to the renal arteries) by abdominal ultrasound or by direct visualization at necropsy. Dog characteristics, including sex, breed, age, and weight, were recorded. Presenting complaint, physical examination findings, locomotor ability, results of diagnostic tests performed, and initial clinicopathological data were also documented. All diagnostic imaging was performed or reviewed by a board‐certified diagnostic imaging specialist. All laboratory diagnostics were performed at the clinical pathology laboratories supervised by board‐certified clinical pathologists. Assessment of acute and chronic ATh was determined based on chronicity of signs. Dogs showing signs for at least 48 hours were considered chronic, and dogs showing signs for less than 48 hours acute. Underlying conditions possibly contributing to ATh in individual dogs were recorded after review of the medical records. Dogs were classified as cryptogenic in the absence of a concurrent diagnosis of a medical condition known to predispose to a hypercoagulable state. In‐hospital and outpatient treatment, overall duration of hospitalization, survival to discharge, and 180‐day survival were recorded when available.

Descriptive statistics were used to describe dog characteristics and treatment administered. Normality was assessed by the Shapiro‐Wilk test and parametric data are reported as mean (SD) and nonparametric data as median (range). Comparisons between groups (ie, acute and chronic ATh, survivor and nonsurvivor) was tested using a Student's *t* test for normally distributed data, and a Wilcoxon rank sum for nonparametric data. Categorical data were tested with a *χ* ^2^ test or Fisher's Exact test (if more than 2 cell counts were less than 5). The following candidate clinical variables were entered into a backward regression analysis: ambulatory status, BUN, sex, corticosteroid administration, hospitalization, pelvic limb deficits, bilateral clinical signs, and neoplasia. Prediction of outcome was tested with the Wald test with respect to variables' association with survival‐to‐discharge. Significance was set at a *P* < .05. All statistics were performed using commercially available software (SAS v9.3, SAS Institute, Cary, North Carolina; MedlCalc v18.5, MedCalc Software bvba, Ostend, Belgium).

## 3 RESULTS

From the medical records at TCSVM and OSU‐CVM, a total of 100 dogs with ATh were included (61 and 39 dogs, respectively). Aortic thrombosis was diagnosed by ultrasonography in 91 cases and by direct visualization at necropsy in 9 cases. Dogs diagnosed with ATh during necropsy did not have an ultrasound performed but exhibited clinical signs compatible with ATh.

### 3.1 Demographics

There were 47 spayed female dogs, 40 castrated males, and 13 intact male dogs. The median age was 10 years (2‐17 years). Median body weight was 24.7 kg (3.2‐45.1 kg). Thirty‐six breeds were represented including: Labrador retriever (n = 16), Greyhound (n = 14), Shetland sheepdog (n = 7), mixed breed dog (n = 7), Border collie (n = 5), Standard Poodle (n = 3), Beagle (n = 3), Rottweiler (n = 3), Golden Retriever (n = 3), Pug (n = 3), Weimaraner (n = 3), Maltese (n = 2), Staffordshire Bull Terrier (n = 2), Pit bull (n = 2), Cavalier King Charles Spaniel (n = 2), Chihuahua (n = 2), Dalmatian (n = 2), and Cocker Spaniel (n = 2).

### 3.2 Physical examination at admission

For all cases, the median rectal temperature was 101.2°F (96.8‐105.1°F), median heart rate was 120 beats/min (50‐220), median respiratory rate was 32 breaths/min (12‐70), and the median systolic blood pressure was 162 mm Hg (80‐240). Sixty‐two dogs had a Doppler blood pressure > 160 mm Hg. Pelvic limb physical examination abnormalities were identified in 85/100 dogs; 35/85 were nonambulatory at the time of presentation, 32/85 were considered painful, 62/85 had bilateral locomotor abnormalities, 56/85 had absent femoral pulses bilaterally, and 30/85 were noted to have cold pelvic limbs.

### 3.3 Onset of clinical signs

The median duration of clinical signs before presentation was 10.5 days (0‐912). Twenty‐seven dogs were presented with acute signs, 72 with chronic signs, and 1 dog with an unknown duration of clinical signs. Dogs with acute signs were more likely to be nonambulatory (*P* < .001), have pelvic limb neurologic deficits (*P =* .007), have bilateral signs (*P =* .01), and be painful (*P =* .03) compared to dogs with chronic ATh (Table 1). Dogs with an acute onset of signs were significantly less likely to survive to discharge (*P* = .04). However, the percentage of euthanasia‐at‐admission was similar for dogs with acute signs compared to dogs with chronic signs (22% and 18%, respectively; *P* = .41).

**TABLE 1:** Summary of statistically significant presenting data between dogs with an acute (<48 hours) vs chronic (>48 hours) development of clinical signs; *P* values <.05 were considered statistically significant

	Acute patients	Chronic patients	*P* value
Nonambulatory	63%	25%	<.001
Neurologic deficits	96%	71%	.01
Pain	50%	26%	.02
Bilateral signs	85%	39%	.01

### 3.4 Clinicopathological and diagnostic imaging data

Common clinicopathologic abnormalities included aspartate transaminase (n = 50), ALP (n = 42), creatine kinase (n = 36), BUN (n = 38), creatinine (n = 21), bilirubin (n = 20), and alanine transaminase (n = 41) above the reference interval (RI) for the laboratory; and albumin (n = 31), hematocrit (n = 21), and protein (n = 20) below the RI (Table 2). Urine dipstick protein estimation was performed in 69 dogs and revealed severe (3+) proteinuria in 51% of these dogs. Urine protein creatinine ratio (UPCR) was subsequently performed in 27 dogs; the median UPCR was 5.2 (0.3‐30.2).

**TABLE 2:** Complete blood count and serum chemistry results from 100 dogs with ATh

Variable	All	Survivor	Nonsurvivor	Number of dogs	*P* value
Packed cell volume (%)	44.47 (±11)	44.1 (±11)	44.9 (±11)	86	.98
White cell count (k/μL)	14.95 (5.86‐72.8)	13.5 (5.86‐72.8)	15.8 (7.4‐65.1)	86	.1
Platelet count (k/μL)	220 (14‐618)	222.5 (14‐618)	218 (40‐494)	85	.7
Total protein (g/dL)	6.18 (±1.32)	6.25 (±1.3)	6.07 (±1.4)	75	.57
Albumin (g/dL)	3 (1.1‐4.1)	3.0 (1.1‐4.1)	3.0 (1.4‐4.0)	89	.98
BUN (mg/dL)	28 (9‐238)	30 (9.0‐238)	25 (10‐136)	91	.27
Creatinine (mg/dL)	1.3 (0.5‐18.5)	1.5 (0.5‐18.5)	1.05 (0.7‐11.3)	91	.15
Total bilirubin (mg/dL)	0.2 (0.08‐5.1)	0.2 (0.08‐2.69)	0.2 (0.1‐5.1)	85	.22
ALT (U/L)	115 (18‐2768)	100.5 (18‐1556)	156.5 (25‐2768)	86	.45
AST (U/L)	167 (22‐7917)	125 (22‐7917)	218 (22‐4056)	84	.25
ALP (U/L)	154 (13‐14 607)	128 (13‐5875)	244 (20‐14 607)	86	.14
Cholesterol (mg/dL)	282 (129‐553)	281.5 (129‐553)	282 (185‐536)	39	.54
CK (U/L)	1093 (63‐417 760)	521 (63‐417 760)	3154 (92‐63 720)	68	.25
AT	75 (±25)	79 (±24)	67 (±28)	36	.2
Thromboelastrogram *G* value^a^	9.8 (±5.6)	9.2 (±6.0)	11.6 (±5.0)	21	.43

*Note:* Parametric variables are presented as mean (±SD) whereas nonparametric variables are reported as median (range).

Abbreviation: ATh, aortic thrombosis; ALP, Alkaline phosphatase; ALT, Alanine transaminase; AST, Aspartate transaminase; AT: anti‐thrombin; BUN, blood urea nitrogen; CK, creatine kinase.

^a^ Reference range for *G* values was 3.1 to 10.0 for OSUCVM for non‐Greyhound patients, 2.5 to 6.2 for OSUCVM Greyhounds, and 4.6 to 10.9 for TCSVM.

Prothrombin time (PT) was measured in 43 dogs (28 survived to discharge and 15 nonsurvivors). Prothrombin time was above the upper end of the RI for the specific machine used in 11 dogs. Out of the 11 dogs with prolonged PT, 2 survived to discharge and 9 were nonsurvivors (*P* < .001). Activated partial thromboplastin time was measured in 47 dogs (27 survived to discharge and 20 nonsurvivors) and was above the upper end of the RI in 9 dogs. Thromboelastrography (TEG) was performed in 21 dogs; 11 dogs were considered hypercoagulable, 7 dogs normocoagulable, and 3 dogs hypocoagulable. We defined a hypercoagulable TEG tracing in our study as having a clot strength G above the breed‐specific reference ranges for each institution, as previously described in dogs.^26^, ^27^, ^28^ Hypocoagulable tracings were defined as having a clot strength G below the breed‐specific RI's for each institution. Mean plasma Anti‐thrombin (AT) activity was 75% (±25). Dogs with protein‐losing nephropathy (PLN) had a mean AT activity of 64% (±27) and those without a PLN had a mean AT of 82% (±22; *P* = .04). The thrombus was located at the terminal aorta in 99 dogs (1 dog had a thrombus located mid‐abdominally), with concurrent aorto‐iliac thrombosis being noted in 54 dogs. An assessment of the occlusive characteristics of the thrombus was recorded in 90/100 dogs, with complete occlusion recorded in 10/90 dogs, partial occlusion in 64/90 dogs, and 16/90 were nonocclusive. Echocardiography was performed or supervised by a board‐certified cardiologist in 35/100 dogs. The main findings were suspected infective endocarditis (n = 4), a visible thrombus within the left ventricle (n = 3), mitral insufficiency (n = 3), left ventricular hypertrophy (n = 3), cardiac mass (n = 3), main pulmonary artery thrombus (n = 1), and aortic insufficiency (n = 1). Dogs whose echocardiograms identified thrombi in the left ventricle were not diagnosed with concurrent structural heart disease based on echocardiography findings. None of those dogs were considered to have a cardiogenic cause of ATh, as dogs with endocarditis and cardiac masses were classified as infectious and neoplastic, respectively.

### 3.5 Possible causes of ATh

The most commonly diagnosed systemic condition known to be associated with hypercoagulability in our study population was PLN (n = 32), either alone (n = 22) or associated with 1 (n = 8) or 2 other conditions (n = 2; Table 3). Neoplasia was diagnosed in 22 dogs, either alone (n = 11) or associated with 1 (n = 8) or 2 other conditions (n = 1). Exogenous corticosteroid use was documented in 16 dogs, mainly associated with 1 (n = 8) or 2 other conditions (n = 3). An endocrine disease was diagnosed in 13 of dogs, including hyperadrenocorticism (n = 9), diabetes mellitus (n = 2), or hypothyroidism (n = 2), either alone or associated with other conditions. Infectious causes were identified in 9 dogs including endocarditis (n = 3), urosepsis (n = 2), anaplasmosis (n = 1), or in conjunction with another illness (n = 3). Overall, 54 dogs were diagnosed with 1 condition associated with a hypercoagulable state, and 23 had 2 or more conditions associated with a hypercoagulable state. It is suspected that the presence of a hypercoagulable state might have contributed to ATh development.

**TABLE 3:** A summary of all diagnoses made in dogs with ATh

Single condition	Number of dogs	Multiple conditions	Number of dogs
Cryptogenic	23	PLN + exogenous steroids	3
PLN	22	PLN + neoplasia	2
Neoplasia	11	PLN + hyperadrenocortiscism	2
Exogenous steroid use	5	Neoplasia + recent surgical intervention	2
Hepatopathy	4	Hyperadrenocorticism + neoplasia	2
CKD	3	PLN + diabetes mellitus	1
Endocarditis	3	Hyperadrenocorticism + exogenous Steroids	1
Hyperadrenocorticism	2	Neoplasia + hypothyroidism	1
Urosepsis	2	Hepatopathy + exogenous steroids	1
Anaplasma infection	1	Urosepsis + hepatopathy	1
Diabetes mellitus	1	Neoplasia + exogenous steroids	1
		Hyperadrenocorticism + urosepsis	1
		Endocarditis + exogenous steroids	1
		Pancreatitis + exogenous steroids	1
		PLN + exogenous steroids + neoplasia	1
		PLN + exogenous steroids + hypothyroidism	1
		Hyperadrenocorticism + exogenous steoids + CKD	1
TOTAL	77		23

*Note:* Single diagnoses are listed in the left column and multiple diagnoses are listed in the right.

Abbreviations: ATh, aortic thrombosis; PLN, protein‐losing nephropathy; CKD, Chronic kidney disease.

Twenty‐three dogs were classified as cryptogenic despite diagnostic testing including an abdominal ultrasound (n = 16), a complete blood count (n = 15), a chemistry profile (n = 15), a urinalysis (n = 15), an echocardiogram (n = 10), PT/pro‐thrombin time (n = 9), and/or a TEG (n = 4). Only 6/23 did not have any diagnostics performed and were euthanized upon admission. The mean age of cryptogenic dogs was 10.7 ± 2.8 years. The median body was 28.7 kg (5.9‐44.0) and 56 were males. Twelve different breeds were represented, and the most common was Greyhounds (n = 6). There was no statistically significant between the percentage of Greyhounds in the cryptogenic group (26%) vs the noncryptogenic groups (10%; *P* = .08). Cryptogenic dogs survived to discharge at a rate of 60.1% compared to 55.8% from noncryptogenic dogs (*P* = .81).

### 3.6 In‐hospital treatment

**FIGURE 1:**
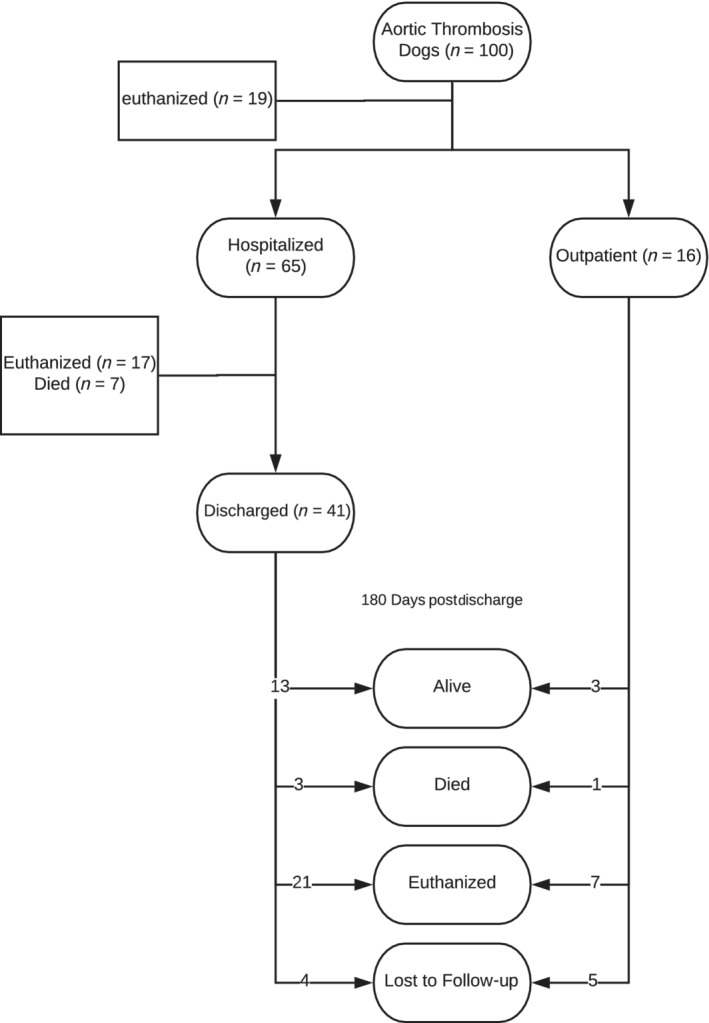
Summary of outcomes for dogs with Ath. Once diagnosed, dogs either were admitted for hospitalization, euthanized or discharged. Of those not admitted, 19 dogs were euthanized and 16 were discharged, with 3 of these 16 being alive 180 days later. Of dogs that were hospitalized, 17 were euthanized, 7 died and 41 were discharged with 13 of these 41 being alive 180 days after discharge

Sixty‐five dogs were hospitalized for in‐hospital management (Figure 1). The median duration of hospitalization was 2 days (0‐12). In‐hospital treatment included a platelet inhibitor, either clopidogrel (n = 28), aspirin (n = 22), or both (n = 2). Unfractionated heparin was prescribed in 27 dogs, by intermittent bolus (n = 23) or by constant rate infusion (n = 4). Low molecular weight heparin was administered in 14 dogs, using dalteparin (n = 11) or enoxaparin (n = 3). Warfarin was used in 8 dogs and rivaroxaban was used in 1 dog. Thirty‐one dogs received multiple thromboprophylatic agents. Thrombolysis was performed using systemic streptokinase (n = 5), local TPA infusion (n = 3), systemic TPA (n = 2), or systemic urokinase (n = 2). Nine dogs had thrombectomy performed via balloon catheterization. Hirudotherapy with medical leeches was prescribed in 2 dogs. Three dogs had pelvic limb amputation during hospitalization, due to concerns about tissue viability in the affected limb.

### 3.7 Outcome

A total of 57 dogs survived to hospital discharge including the 16 dogs treated as outpatients and 41 of the hospitalized dogs (Figure 1). The only variable significantly correlated with survival to discharge in our regression analysis model was the ambulation status, with a Wald *χ* ^2^ of 12.9 (*P* < .001).

Survival to 180 days after discharge was available for 48 cases at which time 16 dogs were still alive, 32 were deceased. Nine dogs were lost to follow‐up. Out of the 48 cases with 180 days survival data available, 14 were discharged without treatment, and the 34 remaining dogs were discharged with an antiplatelet drug monotherapy (n = 15), anticoagulant drug monotherapy (n = 5), combined anticoagulants and antiplatelet medications (n = 12), or combined anticoagulant drugs (n = 2; Table 4). Association between treatment and 180‐day survival was not investigated because of heterogeneity of treatments.

**TABLE 4:** Summary of thromboprophylaxis treatments prescribed following discharge from hospital separated by 180‐day survival

Treatment	All (n = 48)	Alive at 180 days (n = 16)	Dead at 180 days (n = 32)
Antiplatelet monotherapy	Aspirin (n = 8), clopidogrel (n = 7)	Aspirin (n = 3), clopidogrel (n = 4)	Aspirin (n = 5), clopidogrel (n = 3)
Anticoagulant monotherapy	UH (n = 1), LMWH (n = 2), warfarin (n = 2)	Unfractionated heparin (n = 0), low molecular weight heparin (n = 1), Warfarin (n = 0)	Unfractionated heparin (n = 1), low molecular weight heparin (n = 1), warfarin (n = 2)
Anticoagulant + antiplatelet agents	Aspirin + UH (n = 2), aspirin + LMWH (n = 2), aspirin + warfarin (n = 1), aspirin + warfarin + UH (n = 1), clopidogrel + UH (n = 3), clopidogrel + LMWH (n = 3)	Aspirin + UH (n = 1), clopidogrel + LMWH (n = 2), Riva + LMWH (n = 1)	Aspirin + UH (n = 1), aspirin + LMWH (n = 2), aspirin + Warfarin (n = 1), aspirin + warfarin + UH (n = 1), clopidogrel + UH (n = 3), clopidogrel + LMWH (n = 1)
Multiple anticoagulant	UH + LMWH (n = 1) rivaroxaban + LMWH (n = 1)	UH + LMWH (n = 1)	
No treatment	n = 14	n = 3	n = 11

*Note:* This table does not include the 9 dogs that were lost to follow‐up at 180 days.

Abbreviations: LMWH, low‐molecular weight heparin; UH, unfractionated heparin.

## 4 DISCUSSION

The major findings in our study highlight the difference between aortic ATh/thromboembolism in dogs and cats. We were able to identify ambulatory status as the only predictor of outcome. We also found that primary cardiac disease was rarely the sole contributing factor to ATh development. Rather PLN was the most commonly diagnosed condition known to generate a hypercoagulable state, potentially predisposing to ATh formation. Our study population consisted predominately of older, mid to large breed dogs with slightly more males than females and was comparable with prior studies.^1^, ^2^, ^3^, ^6^ Only 2 dogs were Cavalier King Charles Spaniels and 7 were Shetland sheepdogs though those 2 breeds are identified as being commonly affected by ATh.^1^, ^3^, ^6^ Our study population included a high number of Greyhounds (n = 14) relative to other breeds, which might be attributed to either a breed predisposition, or can be related to a large Greyhound referral population in 1 of the included centers (OSUCVM).

The main clinical signs associated with ATh in our study were chronic, mild locomotor abnormalities, and neurologic deficits, which is consistent with previous studies in dogs with ATh,^1^, ^2^, ^3^, ^6^ but differs from the clinical syndrome in cats, where acute, severe paralysis is seen.^4^, ^18^, ^19^ Specifically, 64% of the dogs in our study were ambulatory, slightly less than the 81% to 84% reported by other studies.^1^, ^6^ Consistent with other studies, only 56% of dogs were documented as having absent femoral pulses.^1^, ^6^ Therefore, the presence of femoral pulses do not rule out an ATh, as both incomplete vessel occlusion and the presence of collateral circulation can explain this finding.^18^, ^29^ Seventy‐two percent of dogs in our study were classified as having chronic ATh, defined in our study as an onset in clinical signs beginning ≥48 hours before presentation. Similarly, previous studies reported a 48% to 69% as chronic, though definitions of chronicity varied from more than 24 hours^2^, ^6^ to greater than 2 weeks.^1^ The substantial proportion of chronically affected ATh dogs is in line with the hypothesis that ATh in dogs arises from in situ thrombus formation, manifesting as a gradual onset of clinical signs in contrast to the thromboembolic disease seen in cats.^1^, ^2^, ^29^ However, as clinical signs were more frequent and severe in acutely affected dogs, a small subset of dogs with ATh might experience a true thromboembolic event leading to acute, severe paralysis, which also has been described in that species.^30^

Protein losing nephropathy was the most commonly diagnosed condition known to generate a hypercoagulable state in our study, with 32% of dogs affected, consistent with other studies where PLN accounts for 10% to 35% of cases.^1^, ^2^, ^6^, ^31^ Thus, there might be a relationship between the hypercoagulable state created by this disease and the development of ATh. One proposed mechanism for coagulopathy resulting from PLN is loss of AT, with 1 study documenting low AT activity in 12.5% of PLN dogs.^32^ Dogs with PLN in our study did have significantly lower AT than those without a PLN. Another study reported low AT (<70%) in only 26% of dogs with PLN with 89% of this same population considered hypercoagulable on TEG,^33^ indicating that other factors beyond AT loss contribute to the hypercoagulable state seen in PLN dogs. Mechanisms of hypercoagulability in dogs with PLN need to be further investigated. In our study, a similar comparison cannot be drawn as only 3 dogs with PLN had both AT and TEG values reported. Other commonly diagnosed diseases possibly associated with ATh in our study included neoplasia, endocrine disorders (hyperadrenocorticism, hypothyroidism, diabetes mellitus), and infection. These diseases have been previously implicated in dogs and people^9^, ^10^, ^12^, ^13^, ^31^, ^34^, ^35^, ^36^, ^37^, ^38^, ^39^, ^40^ in the development of thrombosis by developing a hypercoagulable state.^37^, ^41^, ^42^ Sixteen dogs in our study were receiving exogenous corticosteroids at the time of presentation, with 5 dogs having no other risk factor predisposing to ATh. Exogenous administration of corticosteroids increases risk for deep venous thrombosis (DVT) in humans^43^, ^44^, ^45^, ^46^ and has been identified as a possible risk factor for ATh in dogs.^1^, ^2^, ^3^, ^7^ Although previous studies have proposed a possible association between heart disease and the development of ATh in dogs,^3^, ^6^, ^7^ none of the dogs in our study were diagnosed with a structural heart disease thought to be the cause of the ATh, consistent with another recent study where structural heart disease was not identified in any of the 26 ATh dogs studied.^1^ However, 4 of our dogs had evidence of infective endocarditis on echocardiogram and 3 had a cardiac mass, so it is possible that some types of cardiac disease might contribute to the development of ATh in dogs. However, in contrast to ATE in cats, that role appears to be minimal.

A non‐negligible percentage of the dogs in our study were classified as cryptogenic, consistent with other studies.^1^, ^25^ Most of those dogs underwent an extensive medical workup. Possible explanations include missed or misdiagnosed diseases predisposing to ATh during the medical investigation or inappropriate workup to find the actual cause of the ATh. Moreover, it is also possible that breed‐specific hemostasis characteristics (eg, Greyhounds) might have played a role in ATh development.

Approximately two‐thirds of dogs in our study were treated for ATh, and the treatments were variable, reflecting individual clinicians' preferences. The optimal treatment for ATh is unknown. In ambulatory dogs with a chronic history of ATh, some authors advise medical management using a combination of antiplatelet and anticoagulant medications alongside physical therapy and supportive care.^29^ For acutely affected dogs that are severely affected, interventional techniques have been recommended and are described elsewhere.^29^ The American College of Veterinary Emergency and Critical Care Consensus on the Rational Use of Antithrombotics in Veterinary Critical Care (CURATIVE) guidelines address a clinical case of an ATh dog with PLN. Recommendations for that case are to prescribe antithrombotic therapy with antiplatelets, although anticoagulant might also be effective. The guidelines note that there is insufficient evidence to make a strong recommendation between clopidogrel and aspirin, and that antiplatelet/anticoagulant combination therapy might be considered. The guidelines recommends lifelong therapy^47^ . In humans, there is no definitive evidence as to the antithrombotic therapy to be selected or the appropriate duration of such treatment in the case of ATh and recommendations for treatment are often based on recommendations for the treatment of other types of thrombosis (eg, DVT, pulmonary embolism, ischemic stroke).^48^ Platelet inhibitor drugs were the most common treatment in our study. The rationale for the use of platelet inhibitors is that arterial thrombosis occurs in a high‐shear stress environment that promotes platelet hyper‐reactivity.^49^ Historically, aspirin has been used to minimize the development of recurrence of thromboembolism in ATE in cats. This treatment has fallen out of favor because clopidogrel is a more effective medication to inhibit platelets, and to delay or prevent a relapse of ATE when compared to aspirin.^16^, ^50^ Clopidogrel is also recommended over aspirin for people with acute ischemic stroke.^51^ As clopidogrel is the current antiplatelet drug of choice for ATE in cats and has been suggested to be more effective than aspirin in ATh in dogs, use of clopidogrel to treat ATh in dogs seems a reasonable choice.^47^ Two dogs in our study received both aspirin and clopidogrel. Dogs in 1 study reported clinical improvement and resolution of clinical signs associated with ATh when prescribed both drugs, although clinical signs progressed in the 2 other dogs receiving this drug combination.^2^ Concurrent use of aspirin and clopidogrel has been tenuously associated with increased risk for intracranial bleeding in humans and is not recommended for treating peripheral arterial disease.^52^ Anticoagulant medications were the second most common therapeutic group prescribed (n = 50). Warfarin, a vitamin K antagonist, was used in 8 dogs, in contrast to a previous study where warfarin was used in the majority (54%) of dogs, all of whom showed some improvement of clinical signs, with 5 showing complete resolution.^1^ However, warfarin has fallen out of favor in veterinary medicine because of difficulties in variable dose‐response, therapeutic monitoring, and owner compliance.^53^ It is not currently recommended by the CURATIVE guidelines in a clinical scenario of a dog with ATh due to PLN.^15^, ^47^ Currently, oral anticoagulants (ie, rivaroxaban, apixiban) have become more common in veterinary medicine because of their ease of administration, predictable pharmacokinetics, and superior effects in treating deep vein thrombosis in humans.^29^, ^54^ Thrombolysis was attempted in 12 dogs. A previous study reported 4 dogs with ATh who received systemic TPA, 3 of whom were either died or euthanized due to failure to improve or progression of disease.^1^ One case report describes successful decrease in the size of a catheter‐associated jugular vein thrombus using TPA.^24^ Although the use of TPA and streptokinase in ATE cats has been controversial due to concern for reperfusion injury,^18^, ^19^, ^21^, ^22^ these complications arise with equal proportions when receiving TPA or not when TPA was administered within 6 hours of clinical presentation.^20^ Being presented to a referral center in that timeframe would be an unusual clinical presentation in dogs. In people experiencing an acute ischemic stroke, TPA administration within 6 hours of the onset of clinical signs is recommended and improves functionality and quality of life for at least 18 months after the event.^21^ If a clear indication for thrombolysis is better elucidated in cats with ATE, it might become more common in the treatment of ATh in dogs. Nine dogs underwent thrombectomy via balloon catheterization. This technique has been described in dogs, as well as additional methods including catheter and surgical thrombectomy.^29^, ^55^ In people with an acute ischemic stroke, thrombectomy is typically not recommended due to an increased risk of death in the face of a similar rate of good clinical outcome when compared to medical management.^51^ Local delivery via catheter directed thrombolysis is used in humans with severe cases of DVT^56^ and has been described in 1 dog with ATh using TPA.^55^ It is currently unknown if these same guidelines will be translatable to small animals.

Our study showed a discharge rate of 57%, with 28% of discharged dogs alive at 180 days. Our study identified ambulatory status as the only predictor of outcome. This finding might be related to owners being willing to pursue treatment on less affected dogs, and therefore might not be related to animal characteristics or treatment instituted. Our study did not identify chronicity as a risk factor for death, contrary to a previous study that documented a difference in median survival times between acute and chronic (ie, clinical signs >24 hours) dogs (9 days vs 293 days, respectively).^2^ This difference might be explained by difference in severity of disease, or long‐term impact of therapy instituted. Our survival rate is difficult to compare to other studies, as identification of time of death (ie, hospital discharge or final outcome) is lacking in the 2 major retrospective studies.^1^, ^2^

Our study has several limitations. As a retrospective study, dogs were not randomized to treatment, and the treatments used varied greatly in the medications, dosing, and outpatient protocols. Some data points were missing, including more thorough TEG evaluation, and some information such as adverse effects of treatment or clot resolution was not collected. Case selection was based on ultrasound and necropsy findings, and it is possible that some cases of ATh in dogs were not included because of the use of another diagnostic modality. Dogs with ATh might have been underdiagnosed in dogs with mild locomotor or neurological abnormalities because of the lack of recognition and clinical suspicion. As all cases were collected from specialty centers, it is possible that our study population reflects the most severely affected ATh dogs. The cases collected from each of the centers span different timeframes within the period of our study and might reflect an evolving understanding of thrombosis over the 17‐year span of our study. In addition, our logistic regression was determined using several candidate variables chosen specifically by the authors. The variables chosen could be derived from medical records and assessed in isolation. Consequently, some potentially important disease characteristics (eg, chronicity of clinical signs) might have been overlooked in our regression modeling.

## CONFLICT OF INTEREST DECLARATION

Authors declare no conflict of interest.

## OFF‐LABEL ANTIMICROBIAL DECLARATION

Authors declare no off‐label use of antimicrobials.

## INSTITUTIONAL ANIMAL CARE AND USE COMMITTEE (IACUC) OR OTHER APPROVAL DECLARATION

Authors declare no IACUC or other approval was needed.

## HUMAN ETHICS APPROVAL DECLARATION

Authors declare human ethics approval was not needed for this study.
